# Low Molecular Weight Hydrogel for Wound Healing

**DOI:** 10.3390/pharmaceutics15041119

**Published:** 2023-03-31

**Authors:** Shangyan Gu, Yu Lu, Yuji Wang, Wensheng Lu, Wei Wang

**Affiliations:** 1 Centre for Pharmacy, University of Bergen, 5020 Bergen, Norway; 2 School of Pharmacy, Capital Medical University, Beijing 100069, China; 3 Institute of Chemistry, Chinese Academy of Sciences, Beijing 100190, China; 4 Department of Chemistry, University of Bergen, 5020 Bergen, Norway

**Keywords:** supramolecular hydrogel, wound healing, C18ADPA, copper, self-assembling

## Abstract

Octadecylazanediyl dipropionic acid (C18ADPA) is a zwitterionic amphiphile with a dendritic headgroup. C18ADPA self-assembles to lamellar networks, which encompasses water and forms a low-molecular-weight hydrogel (LMWG). In this study, we use the C18ADPA hydrogel as a drug carrier for the in vivo delivery of a copper salt for wound healing in a mouse model. A structural transition was observed based on cryo-scanning electron microscope (cryo-SEM) images after drug loading. The C18ADPA hydrogel, which had a layered structure, transformed into a self-assembled fibrillar network (SAFiN). The mechanical strength of the LMWG has always been an important issue in its applications. However, due to the structural transition, both the storage and loss moduli increased. In vivo tests showed that wound closure was faster after applying the hydrogel formulation compared with the Vaseline formulation. For the first time, we have also provided histological evidence of these effects on skin tissue. The hydrogel formulation exhibited clear advantages in regenerating tissue structure over traditional delivery formulations.

## 1. Introduction

Hydrogels are frequently employed in drug formulation to achieve controlled release owing to their high fractal resistance and tortuosity [1]. The three-dimensional structure of hydrogels restrains the motion of drug molecules, thereby controlling the release rate through the regulation of the diffusion process [2]. Hydrogels are widely used in various dosage forms as a moderator for sustained release. For instance, Carbopol^®^, a hydrogel family made from polyacrylic acid, is used in suspensions, bioadhesive formulations, solid dosage wet granulation, and more [3].

Low molecular weight gel (LMWG), also known as molecular gel, is a novel type of gel [4]. In LMWGs, gelators with molecular weights below 3000 Da self-assemble to form an organized structure via intermolecular interactions [5]. LMWG exhibits some resemblance to the extracellular matrix [6]. Consequently, researchers anticipate promising applications of this material in biological systems [7]. Numerous studies have demonstrated the potential applications of LMWG in tissue engineering [8], enzyme immobilization [9], cell culture [10], and drug delivery [11,12]. LMWG has several advantages as a drug carrier, such as controlled release properties, biocompatibility, and biodegradability. For example, Nilsson and colleagues used N-Fluorenylmethoxycarbonyl (Fmoc) phenylalanine to deliver diclofenac in vivo for anti-inflammatory purposes [13]. Cao et al. demonstrated the in vitro release of salicylic acid from a supramolecular phenylalanine-derived hydrogel [14]. Xu and colleagues used the supramolecular hydrogel composed of pamidronate, Fmoc-Leu, and Fmoc-Lys to reduce inflammation and the toxicity of UO22+ [15]. However, the use of these hydrogels for in vivo drug delivery is problematic due to the poor mechanical stability of the resulting gel system.

Our previous studies have shown that C18ADPA forms a stable hydrogel in a narrow pH range [16,17]. The hydrogel exhibits a reversible bilayer-to-micelle transition through temperature adjustment. In the case of LMWGs, adding a third component can be challenging since gelation is based on the fine balance of supramolecular interactions [5]. Preserving mechanical stability when the third component contains metal ions is even more difficult [18]. In this study, we present an investigation using the C18ADPA hydrogel as a robust drug carrier, even for drugs containing metal ions. Moreover, we report, for the first time, a histological analysis of how the LMWG, as a drug carrier, influences the regeneration of skin tissues. A quantitative assessment of clinical features and histological parameters revealed relatively faster healing using the formulation made from the C18ADPA hydrogel. Compared to traditional Vaseline formulations, the C18ADPA hydrogel offers advantages in all healing phases.

## 2. Materials and Methods

### 2.1. Materials

**Figure 1:**
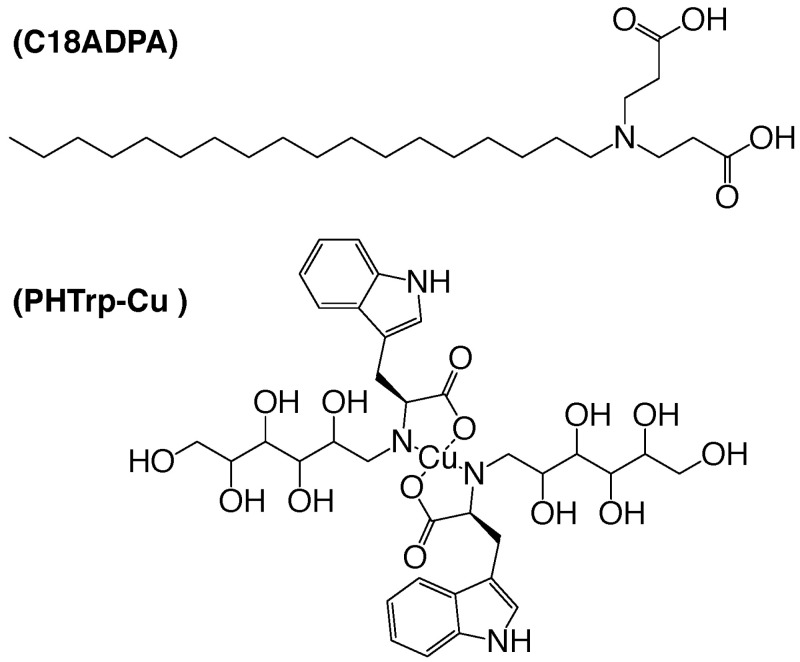
The chemical structures of C18ADPA and PHTrp-Cu.

Methacrylate (>99%) and octadecylamine (>96%) were purchased from Adamas-Beta (Shanghai, China). L-Trp, D-glucose, ethyl carbamate, and *N*-methylmorpholine were purchased from Sigma-Aldrich (St. Louis, MO, USA). Methanol (99.5%), hexane (98%), sodium hydroxide (96%), and hydrochloric acid (36.5%) were used as received from Tianjin Chemical Factory (Tianjin, China). White Vaseline cream was purchased from Aladdin Industrial Corporation (Shanghai, China). Milli-Q water was used for all the experiments. We refer the reader to our previous publications for the synthesis of zwitterionic amphiphile 3,3′-(octadecylazanediyl) dipropionic acid (C18ADPA) (see the chemical structure in Figure 1). The data was analyzed using a two-way ANOVA (GraphPad Prism 9.0).

### 2.2. Preparing N-(2,3,4,5,6-Pentahydroxylhexyl)-L-Trp (PHTrp)

2.04 g (10 mmol) of L-Trp was dissolved in a solution of 0.40 g (10 mmol) of NaOH in 3 mL of methanol/water (1:1). Then, 1.80 g (10 mmol) of D-glucose were added to this solution. Under the protection of argon gas, the mixture was stirred at 55 °C for 6 h to form intermediate 1, which was in-situ reduced by 1.62 g (30 mmol) of NaBH4 at room temperature for 96 h. Then, the mixture was adjusted to pH 2.5 with HCl at 0 °C and precipitates were formed in the solution. The filtrate was concentrated after the precipitation was removed by filtration. The concentrate was diluted with anhydrous ethanol and the insoluble matter was removed by filtration. After repeating this step five times, the residue was dissolved in 10 mL of water. The PHTrp (19% yield, colorless powders) was purified using an acidic ion exchange resin column and was eluted using a 3% aqueous solution of *N*-methylmorpholine. Mp 206–207 °C, $\alpha_{D}^{20}$ = +10.0 (C = 1.6, H_2_O). IR (KBr): 3407, 3353, 3095, 2968, 2916, 1617, 1596, 1400, 1355, 1080, 1042, 742, 675, 534 cm^−1^. ESI (+)/FT-MS (m/e): 369.15620 [M+H]^+^. ^1^H NMR(300 MHz, D_2_O): δ = 7.22 (d, *J* = 7.5 Hz, 1H), 7.20 (d, *J* = 7.5 Hz, 1H), 7.16 (t, *J* = 7.8 Hz, 1H), 7.14 (t, *J* = 7.8 Hz, 1H), 6.92 (s, 1H), 4.16 (m, *J* = 4.8 Hz, 1H), 3.90 (t, *J* = 5.1 Hz, 1H), 3.85 (m, *J* = 5.1 Hz,1H), 3.83 (m, *J* = 5.1 Hz, 1H), 3.77 (m, *J* = 3.9 Hz, 1H), 3.71 (d, *J* = 5.1 Hz, 2H), 3.27 (dd, *J* = 3.6 Hz, *J* = 12.9 Hz, 1H), 3.2 0(dd, *J* = 9.3 Hz, *J* = 12.9 Hz, 1H), 2.93 (d, *J* = 4.9 Hz, 2H).

### 2.3. Preparing the Complex of PHTrp and Cu^2+^ (PHTrp-Cu)

To for a suspension, 368 mg (1.0 mmol) of PHTrp and 170.5 mg (1.0 mmol) of CuCl_2_·2H_2_O were added to 10 mL of water. Then, 40 mg (1.0 mmol) of NaOH was added to form a clean blue solution. The solution was stirred at 60 °C for 10 min and was filtered. The filtrate was purified on size exclusion chromatography (Sephadex G10) to yield 502 mg (92%) of PHTrp-Cu (see Figure 1). ESI^-^/FT-MS (m/e): 500.01675. $\alpha_{D}^{20}$ = +18.5 (C = 1.0, H_2_O). ^1^H NMR(500 MHz, D_2_O) δ = 7.23 (d, *J* = 7.5 Hz, 1H), 7.21 (d, *J* = 7.5 Hz, 1H), 7.18 (t, *J* = 7.5 Hz, 1H), 7.15 (t, *J* = 7.5 Hz, 1H), 6.93 (s, 1H), 4.07 (m, 1H), 3.83 (t, *J* = 5.0 Hz, 1H), 3.75 (m, *J* = 5.0 Hz, 1H), 3.73 (m, *J* = 5.0 Hz, 1H), 3.67 (m, *J* = 3.5 Hz, 1H), 3.61 (d, *J* = 5.0 Hz, 2H), 3.31 (dd, *J* = 3.5 Hz, *J* = 12.5 Hz, 1H), 3.21 (dd, *J* = 9.0 Hz, *J* = 12.5 Hz, 1H), 2.89 (d, *J* = 5.0 Hz, 2H).

### 2.4. Preparation of C18ADPA Hydrogel and PHTrp-Cu-Loaded Hydrogel

C18ADPA (0.2 g) [17,19] and NaOH (0.04 g) were mixed and dissolved in 5 mL of distilled water. The solution was warmed up in a water bath to 70 °C and the mixture gradually became transparent. The pH value was adjusted to 5. In this pH range, the viscosity of the sample underwent a significant change. Then, the tube was removed from the water bath and kept still at room temperature in order to form the C18ADPA hydrogel. In order to prepare a PHTrp-Cu-loaded hydrogel, 5 mL of PHTrp-Cu solution was used instead of distilled water.

### 2.5. Characterization of the Hydrogels

Rheology. The elastic modulus (G’) and viscous modulus (G”) were measured using a TA DHR-1 rheometer (TA instrument GmbH, Newark, NJ, USA) with a plate–plate geometry with a diameter of 40 mm and a default gap of 1 mm. Frequency sweep measurements were carried out in the range of 0.5–100 rad/s in the linear viscoelastic region, determined via dynamic strain sweep measurements.

Differential Scanning Calorimetry (DSC). The DSC measurements were performed using a TA-DSCQ2000 (TA instrument GmbH, Newark, NJ, USA) instrument. The PHTrp-Cu-loaded hydrogel samples (about 20 mg) were sealed into aluminum pans, and the DSC thermo-grams were recorded within a temperature range of 15–65 °C (heating rate = 2 °C min^−1^) under an N_2_ atmosphere.

Cryo-Scanning Electron Microscopy (cryo-SEM). The morphology of the hydrogels was studied using Cryo-SEM high-pressure freezing (Leica, Weztlar, Germany), which prevents or minimizes damage to the hydrogel structure caused by ice crystal formation. The samples (5 µL) were loaded on the cryo specimen holder and were then transferred into a liquid nitrogen bath until the liquid nitrogen ceased boiling. Then, the liquid nitrogen bath was transferred into the vacuum space for immediate vacuuming. The samples were warmed up to −100 °C, and the sample surface was freeze-dried for 30 min by the sublimation of water. The samples were investigated at a temperature of −146 °C and an accelerating voltage of 3 kV or 5 kV. Imaging was performed using the analysis mode and the backscattered electron signal.

X-ray Powder Diffraction (XRD). XRD measurements on the hydrogels were carried out using a Bruker D8 advance diffractometer (Bruker, Billerica, MA, USA). The source of the X-ray was Cu-α radiation with a wavelength of 0.15406 nm. The diffraction pattern was recorded with the diffraction angle in the range of 1°–10°.

Fourier transform infrared spectroscopy (FTIR). FTIR measurements were carried out using a Thermofisher Nicolet 6700 FTIR spectrometer (Thermofisher, Waltham, MA, USA). The samples were prepared by KBr pellets. The scan range of the wavenumber was in the region of 4000–400 cm^−1^.

### 2.6. Animal Test

Healthy male ICR mice were purchased from the Animal Center of Peking University and were used for the experiments. The mice were accommodated in wire topped cages with sterile husk as a bed material and were kept at temperatures between 20–25 °C. Mice were fed with commercial mice feed and water ad libitum. We intraperitoneally injected a 20% solution of ethyl carbamate in order to anesthetize the mice. Each mouse weighted approximately 20 g and was injected with 0.14 mL of the solution. Hair was removed from the dorsum of the mice using an electrical hair remover. The index finger and thumb were used to fold the back at the midline and to lift the dorsal skin in order to form a sandwiched skinfold. Then, the animals were placed in the lateral position, the 5 mm diameter biopsy punch was pressed in order to completely remove the skin layer, and a symmetrical full thickness resection wound was formed. The wound area was assessed and photographed every day until the lesions were fully closed. The ethics committee of Capital Medical University approved all the procedures (the ethics number is AEEI-2018-174). The welfare of all the animals is ensured.

The mice were randomly divided into four groups of at least six mice. Group I (control group) was the group that did not receive any treatment, Group II (matrix group) was treated with 4% C18ADPA hydrogel, Group III (hydrogel group) was treated with PHTrp-Cu-loaded hydrogel, and Group IV (Vaseline group) was treated with PHTrp-Cu-loaded Vaseline as a group for comparison.

The percentage of wound contraction (PWC). The PWC was calculated based on the percentage reduction of the original wound size [20]. The wound area was measured using a digital caliper. The PWC was calculated based on the initial wound area (A_0_) and the wound area on the nth day (A_n_). The PWC was calculated based on the following equation:

(1) $$PWC=\frac{A_{0}-A_{n}}{A_{0}}\times 100\%$$

The animal test was randomized and double-blinded. Data are presented as mean ± SD (standard deviation). The group size was determined on the basis of the results from a preliminary experiment. Statistical significance for all the wound healing studies were determined using the student’s *t*-test. The results were considered statistically significant when *p* < 0.05.

Histology study. Skin tissues were collected on day 2 and day 8. The tissue samples were fixed in 10% formalin solution, dehydrated in a graded alcohol series, starting from 50% and increasing to 75%, 90%, and 100%, cleared in xylene, and embedded in paraffin wax. Then, the tissue sections were stained with Hematoxylin–Eosin (H&E) [21].

## 3. Results and Discussion

### 3.1. Hydrogel Structure

**Figure 2:**
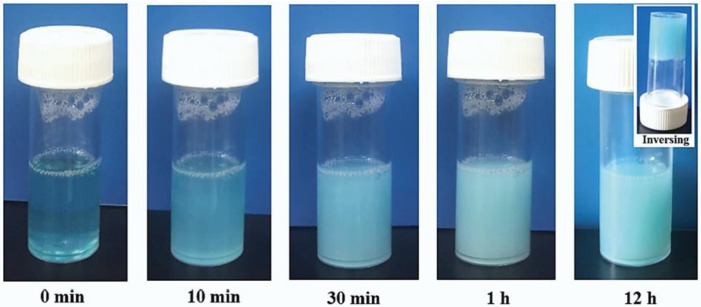
Evolution of the PHTrp-Cu hydrogel.

**Figure 3:**
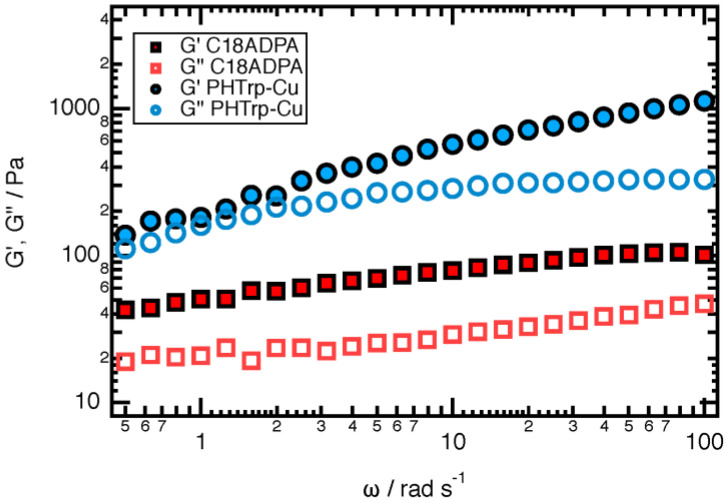
Frequency sweep dynamic rheological data for the C18ADPA hydrogel and the PHTrp-Cu hydrogel.

**Figure 4:**
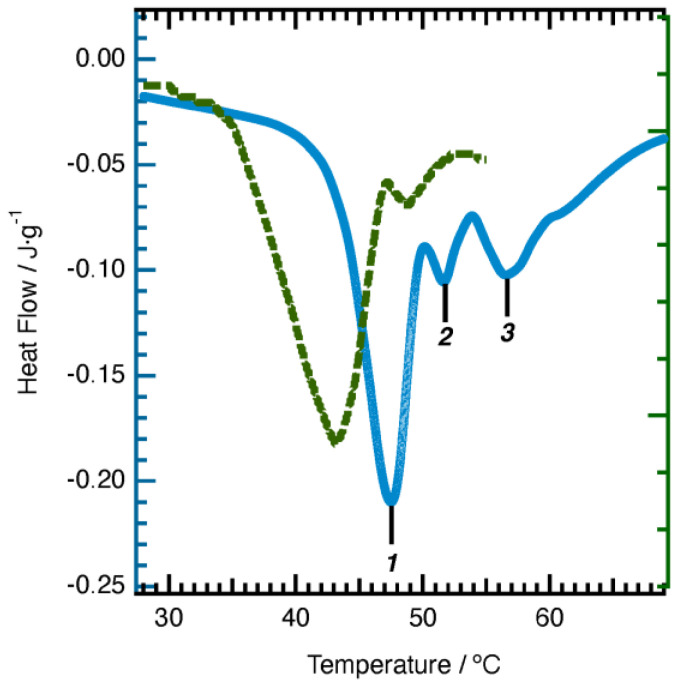
DSC data for the PHTrp-Cu hydrogel. The dotted line represents the data for the C18ADPA hydrogel [17].

A C18ADPA solution forms a hydrogel when the concentration is higher than 2%. The strength of the gel increases with C18ADPA concentration. In order to ensure good mechanical strength for topical use, in this study, all the experiments were conducted with 4% C18ADPA hydrogels. A 4% C18ADPA hydrogel was translucent at 50 °C and turbid at 25 °C. The PHTrp-Cu hydrogel shows the same blue-colored light scattering behavior as the aquo complex of copper (Figure 2). The PHTrp-Cu hydrogel forms very quickly. During cooling at room temperature, the transition of the hydrogel was completed within 30 min. The storage modulus G’ and the loss modulus G” were raised by nearly one order of magnitude once PHTrp-Cu (0.5%) was added to the hydrogel (Figure 3), indicating that the PHTrp-Cu molecules were incorporated into the gel structure and enhanced the mechanical strength of the hydrogel. Examination of the DSC thermogram (Figure 4) confirmed the interaction between PHTrp-Cu molecules and the hydrogelators. The hydrogel without PHTrp-Cu showed a gel-to-gel transition at 43.3 °C (Tg) and an additional endothermic peak at 48.8 °C (Tm) [17]. The corresponding peaks in the PHTrp-Cu hydrogel (Figure 4) were shifted to higher temperatures, 47.6 °C (peak 1) and 51.4 °C (peak 2), respectively. The latter temperature, which was similar to the 49.5 °C observed for the xerogel, was attributed to the melting of the alkyl chains (Tm). In addition to Tg and Tm, another endothermic peak at 56.6 °C (peak 3 in Figure 4) resulted from the interactions between PHTrp-Cu and the hydrogel structure.

**Figure 5:**
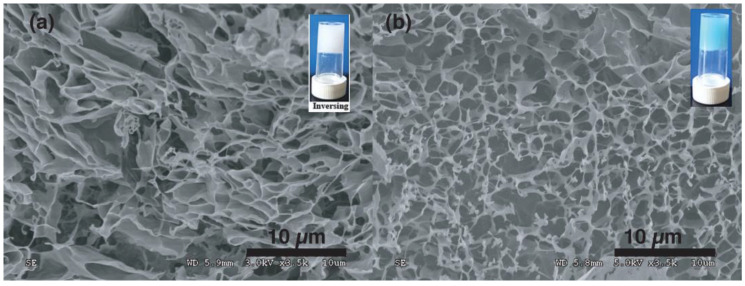
The cryo-SEM images of (**a**) the C18ADPA hydrogel and (**b**) the PHTrp-Cu-loaded hydrogel.

The mesoscopic structure of the hydrogels and the supramolecular packing of C18ADPA with PHTrp-Cu were characterized using cryo-SEM and XRD analysis. For the C18ADPA hydrogel, cryo-SEM clearly revealed the 3D structure; the layered structure interconnected and encompassed the liquid phase in the open spaces (Figure 5a). After loading with PHTrp-Cu, significant changes were observed in the hydrogel structure (Figure 5b). The intact layers of the C18ADPA hydrogel transformed into a fibrillar network in the PHTrp-Cu hydrogel [22]. The self-assembled fibrillar network (SAFiN) traps water molecules through capillary forces. This change on a mesoscopic scale has led to an increase in the mechanical strength of the hydrogel.

**Figure 6:**
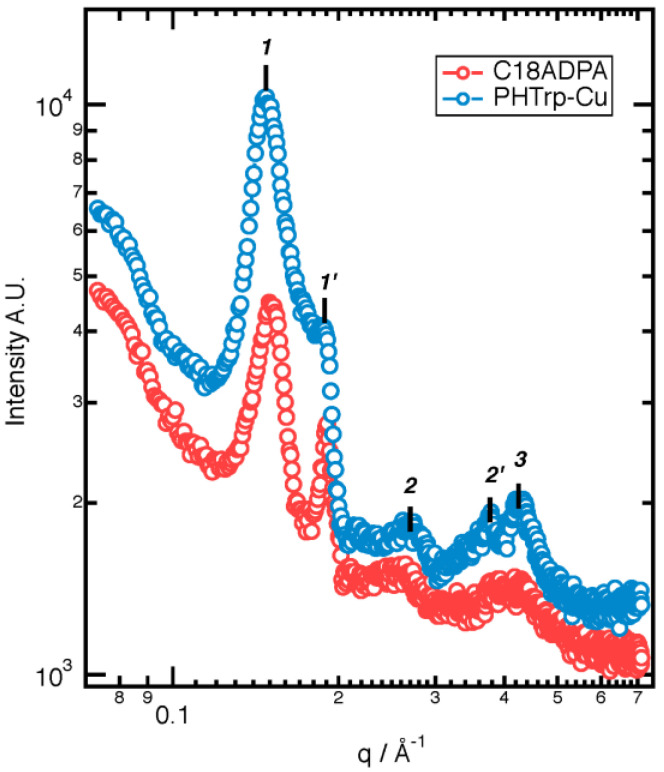
L-XRD data of the C18ADPA hydrogel and the PHTrp-Cu hydrogel.

**Figure 7:**
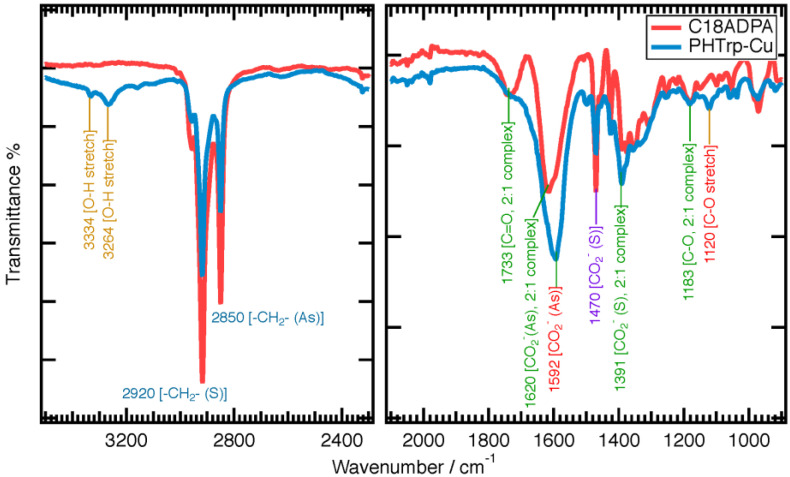
A comparison of the FTIR data of the hydrogels before and after loading PHTrp-Cu. (S) and (As) denote symmetrical and asymmetrical, respectively.

The change in the constructive unit for the PHTrp-Cu hydrogel was rather interesting. Therefore, low-angle XRD (L-XRD) was used to reveal the molecular packing of both the hydrogels (Figure 6). For the C18ADPA hydrogel, the L-XRD data showed the existence of two lamellar structures (1 and 2). The d-spacing of lamellae-1 was d_1_ = 4.1 nm, which is commensurate with the d-spacing observed in (001) reflections of the crystalline lamellar bilayers of octadecyl ammonium crystals [23,24]. Thus, in close connection to the octadecyl ammonium crystals, lamellae-1 consisted of crystalline lamellar bilayers composed of polar and nonpolar layers. As for lamellae-2 (d_2_ = 3.1 nm), a peak was observed at a position very close to that of the d-spacing for the lamellar structure of the xerogel (d = 3.6 nm), which was indicative of the existence of a lamellar structure in which alkyl chains are fully interdigitated [25]. The PHTrp-Cu hydrogel also has the lamellar structure as a building unit for the SAFiN structure. Similar to the C18ADPA hydrogel, two lamellar structures were found and the d-spacing for both lamellae increased. Lamellae-1, with compact packing, has d-spacing of 4.3 nm and lamellae-2, with the interdigitated alkyl chain, has d-spacing of 3.1 nm. The L-XRD results revealed that even though the morphology of the hydrogel changed after adding PHTrp-Cu, mesoscopically, the essential built-up structure was still lamellar. The two lamellar structures correspond very well with the analysis of the DSC results. Since the origin of the two lamellae were due to the crystalline bilayers and the interdigitated alkyl chain, lamellae-1 is then correlated with the endothermic peak of Tm and lamellae-2 is correlated with the endothermic peak of Tg. In order to further analyze the change in C18ADPA after loading PHTrp-Cu, we compared the FTIR spectra (Figure 7) to reveal the interactions between the molecules.

The FTIR spectra was presented in two parts (Figure 7). In the range of 2000–700 cm^−1^, we used a smaller scale of transmittance in order to identify all the peaks. At 3267 cm^−1^, the OH stretch absorption from glucose was identified for the PHTrp-Cu hydrogel sample. The peak was narrow, indicating that the OH groups were in a nonpolar environment [26]. C18ADPA is a zwitterionic surfactant. In the headgroup, the acid groups (–COOH) and the weak base (the tertiary amine) may react and form a complex salt [27]. In the hydrogels, the spectrum of a carboxylic acid:amine 2:1 complex shows bands attributable to this species [28]. The formation of a 2:1 complex indicates that the reaction is effectively complete. It is characterized by a ν(C=O) band at 1733 cm^−1^, a ν(C-O) band at 1183 cm^−1^, ν_s_(CO^2–^) at 1391 cm^−1^, and ν_As_(CO^2–^) at 1620 cm^−1^ for the C18ADPA hydrogel. Three changes were noticed after the addition of PHTrp-Cu: first, the intensity of the ν(C=O) band at 1733 cm^−1^ significantly decreased; second, the ν_As_(CO^2–^) band moved to 1592 cm^−1^; and third, a ν(C-O) stretch band appeared at 1120 cm^−1^. We also observed that the ν(C-O) band at 1183 cm^−1^ was relatively small. Putting this information side-by-side, we realized that the interaction between the headgroups may go through a transition from a 1:2 complex to a 1:1 complex after adding PHTrp-Cu to the hydrogel. The compact arrangement of the three headgroups within the 2:1 complex suggested a smaller effective area of the headgroups compared to the headgroups within the 1:1 complex. This drove the system to adopt a higher curvature self-assembly after adding PHTrp-Cu and forming the SAFiN structure [29].

### 3.2. Animal Test

**Figure 8:**
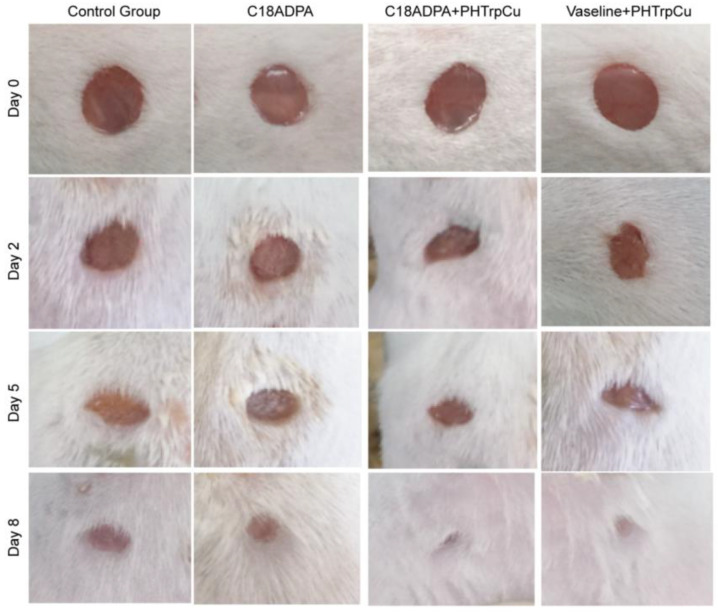
The process of wound healing. The diameter of the wound area was 5 mm on day 0. Pictures were taken on day 0, 2, 5, and 8 for the different groups, and there were six mice in each group.

The hydrogel, as a drug delivery matrix, accelerates wound repair (Figure 8). The same dose of 57 ± 2 mg of PHTrp-Cu was applied using the hydrogel and Vaseline cream once per day for groups III and IV, respectively. The number of mice, the dose, the area of wound spots, and the experimental duration were decided based on a preliminary study. Postoperative observation showed that there was a contraction of the wound area already on the first day. The majority of the wound area had closed by day 7–8, and the remaining area was too small to be precisely recorded. The environment and the drug formulations were not sterile; therefore, we observed small, inflamed areas on day 2 after the operation.

**Figure 9:**
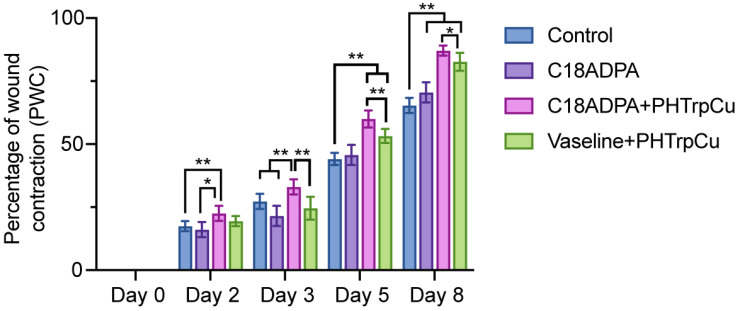
The percentage of wound contraction was calculated based on the results of the animal test (*n* = 6). The data was analyzed using a two-way ANOVA (Prism 9.0). ** *p* < 0.01, * *p* < 0.05.

The wound healing was evaluated by the percentage of wound closure [30]. From day 2, group III (hydrogel formulation) showed a significant reduction in wound area compared to the other groups (Figure 9). The wound closed much faster in the hydrogel group and the Vaseline group. On day 8, these two groups showed an 80–88% reduction in wound area. The order of healing during the whole healing process was as follows: the hydrogel group > the Vaseline group > the matrix group > the control group (Figure 9).

**Figure 10:**
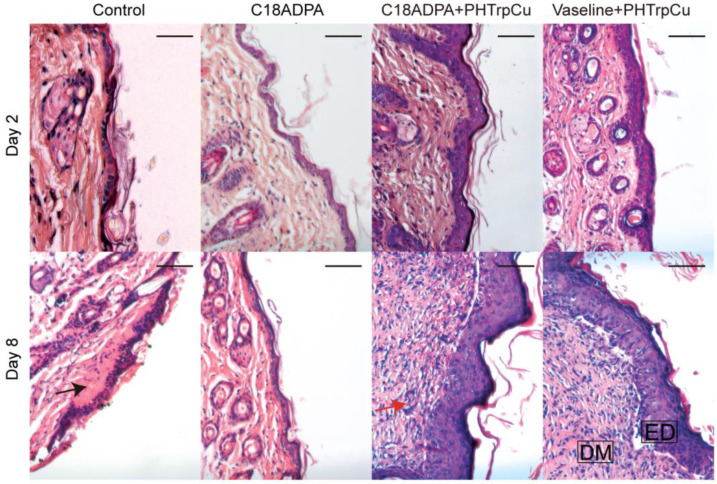
Histological images of wound healing in mice. Images of tissue sections stained with hematoxylin and eosin showing histological changes during the process of wound healing post-injury on day 2 and day 8 for the different groups. Group I is the control group, Group II is the matrix group with the C18ADPA hydrogel, Group III is the group treated with the hydrogel loaded with PHTrp-Cu, and Group IV is the Vaseline group. ED is epidermis; DM is dermis; the black arrows are edema, and the red arrows are hair follicles. Bars represent 200 μm (*n* = 3).

Skin tissues collected on days 2 and 8 were used for the histological analysis (Figure 10). As seen in Figure 10, edema was present with expanded space in the deep dermis and the near wound site for Group I on day 2. The skin edges were healthy and the wound extended to the surface of the deep dermis. The superficial layer of the wound was lined by a thin crusting. Collagen fibers could already be seen from day 2 [31,32]. For the matrix group, edema was still the most predominant feature, but the tissue was less edematous than Group I. Crusting could also be observed in the outer epidermis. Edema and fibrin strands were evident. For Group III, the edema and fibrous appearance resolved significantly. The interstitial layer and the dermis had greater numbers of mononuclear cells. The wound edge with adjacent healthy skin is clearly present. For Group IV, the wound exhibited widespread crusting, and the edges were clearly demarcated. The wound extended to the surface of the deep dermis. Furthermore, the number of hair follicles increased a lot compared to the other groups.

On day 8, for Group I, the wound had widespread crusting and the edges were clearly demarcated. The collagen fibers were thick with a predisposition to form irregular bundles. Widespread crusting can still be found. For Group II, the wound had a low amount of collagen fibers. The wound appeared closed and healed, but was distinct from the adjacent undamaged skin. The inflammatory phase is still the most predominant feature, but it was less edematous. Fibroblasts and new blood vessels were evident. For Group III, the wound had almost completely healed. The keratinocyte layer, fibroblasts, and endothelial cells formed an integral part of the repaired tissue. The collagen fibers at the wound site were very well organized. For Group IV, the wound contracted and the dermis was very well organized in comparison to day 2. The dermis of the wound site had less dense collagen compared to the adjacent healthy dermis. The staining of the healthy side is more distinctly purple–pink due to well organized bundles of collagen.

We compared the histological results and concluded the following:

Epithelialization: The regenerated epidermis could be observed on day 2, and the epithelialization of the wound tissue on day 8 was significantly better than that on day 2. The sign was significantly greater in the hydrogel group and the Vaseline group than in Group I and II.

Vascularization: New vessels formed on day 2 in the treatment groups (Group III and IV), and angiogenesis significantly increased in all the groups on day 8. Compared with the other three groups, the hydrogel group had the highest vascularization score on day 8.

Granulation: On day 2, the epidermis, dermis, and subcutaneous tissues were injured in each group. On day 8, slightly more advanced granulation tissue began to form in the dermis. Among the groups, the C18ADPA hydrogel group with PHTrp-Cu had the thickest granulation tissue area. In contrast, irregular formation of granulation tissue was observed in the Vaseline group with PHTrp-Cu.

Collagen deposition: on day 2, collagen deposition was observed in all the groups. On day 8, a large number of new collagen fibers formed in the dermis. On day 8, collagen regeneration increased significantly in the hydrogel group and the Vaseline group.

## 4. Conclusions

Self-assembly is at the core of many biological transformations. Understanding the principles of self-assembly would help us to create functional materials for biological use. This study is an example of such. We thoroughly studied the structural transition of the C18ADPA hydrogel and its function as a transdermal drug carrier. The structure of the hydrogels was studied on three levels, macroscopically by rheology and DSC, mesoscopically by cry-SEM and L-XRD, and microscopically by FTIR. The SEM images clearly showed the transition from a layered structure to a SAFiN after adding the model drug, PHTrp-Cu. The mechanical strength, which is often a problem for molecular hydrogels, increased in this case. The structural transition was finally confirmed by FTIR spectra with the transition from a 1:2 complex to a 1:1 complex. Most interestingly, the process of wound healing was rather fast, and the skin tissue showed much earlier collagen deposition during regeneration, which may relate to the similar natures of the hydrogel and the biological tissue. We believe that the advantages brought about by this material will open up new, previously unexplored pathways in the application of molecular hydrogels as transdermal drug delivery matrices.

## Data Availability

Data are available upon request.
